# Experimental Study on Long-Term Mechanical Properties of Prestressed Glulam Continuous Beams

**DOI:** 10.3390/ma15124182

**Published:** 2022-06-13

**Authors:** Nan Guo, Shouting Zhou, Yan Zhao, Lidan Mei, Yunan Zhang

**Affiliations:** 1 Department of Civil Engineering, Northeast Forestry University, Harbin 150040, China; snowguonan@163.com (N.G.); zhoushouting0606@163.com (S.Z.); zhangyunan07262021@163.com (Y.Z.); 2 School of Civil Engineering and Architecture, Wuyi University, Wuyishan 354300, China; zhaoyan_hit@163.com

**Keywords:** glulam continuous beam, long-term, creep, deformation coefficient, prestress

## Abstract

To study the effect of wood creep on the long-term performance of prestressed glulam continuous beams, a 180-day test was carried out on beams configured with different numbers of steel wires (2, 4, 6) and with different prestress values (0, 7, 14 kN). By investigating the stress loss of the steel wires in the beam and the change in the mid-span deflection over time, the factors influencing the creep of the continuous beam were analyzed. Three models were selected to fit the creep process of the test beams. Moreover, the creep deformation coefficient *θ* was introduced to reflect the influence of glulam creep on the deflection change in the test beams and to predict the total deflection of the beam within 50 years. The results showed that with increasing the number of steel wires and the prestress value on the beams, the total stress of the steel wires declined more and faster. Increasing the number of steel wires or decreasing the prestress force value could effectively restrain the change speed of the mid-span long-term deflection of the beam. Three models were compared, and the power-law equation was the most accurate. At familiar steel wire quantities and force levels, the *θ* value of the test beams within the design service life of 50 years was determined to be 1.28–2.29.

## 1. Introduction

Timber structure has a long history and is an important element of traditional architectural culture. Various structural systems represented by light-frame timber structures, glued laminated timber structures, and hybrid timber structures have been formed worldwide. In addition, with the signing of the “Paris Agreement” and the policy of “emission peak and carbon neutrality” advocated by China, the research and technology of modern timber structures have gradually expanded. Timber structure has a series of advantages such as green qualities, low energy consumption, livability, and superior seismic properties, and there is a newly developed opportunity in timber-structured buildings [1,2,3,4]. Glulam is a main timber structural material, and its cross-sectional dimensions can be freely specified. Furthermore, glulam has fewer defects and higher available strength and stiffness compared to logs. However, although glulam is widely used, it also has some weaknesses. First, because of the limitations of raw material size and processing technology, the traditional glulam beams generally cannot meet the length requirements for continuous beams or long-span members. Second, most of the simply supported beams suffer brittle tensile failure and larger deformation in the bending state. Therefore, the compressive strength is not fully utilized [5,6,7,8], and deformation is controlled by section size. Third, the traditional glulam joint connection needs grooving and drilling, and the secondary processing will damage the wood at the joints. Therefore, the location of the joint connection is easily cracked along the damaged area under the action of force, the capacity to transfer bending moment is weak, and the joint stiffness is insufficient.

Based on the above problems, our research group proposed the concept of the non-damaged splint-type prestressed glulam continuous beam joint and then developed the joint connection device [9]. According to this connection device, a type of novel composite prestressed glulam continuous beam was proposed. When the continuous beam was bent, the prestressed steel wires were in tension and the glulam was in compression. It incurred ductile failure, like the prestressed glulam simply supported beam. The advantage was that not only was the stiffness of the joint improved through the connection device and prestress and the semi-rigid connection realized, but also the bending moment distribution was changed, to make further full use of the materials and reduce deformation. The research group carried out related research and tested the short-term flexural performance of continuous beams in the early stage [10,11,12]. The creep characteristic of timber is the key factor affecting the long-term mechanical performance of glulam continuous beams, and because of the application of prestressing, the force of the beam is more complicated. Therefore, it is important to investigate the long-term mechanical performance of the prestressed glulam continuous beam and to promote the development of this composite member.

At present, the scholarly research in related fields has mainly focused on the reinforcement of timber members, timber connection, and long-term mechanical performance of timber structures. The timber members are reinforced mainly by adding steel bars, steel plates, and fiber-reinforced materials. In addition, because of the impact on building construction, strength, ductility, and the ability to increase structural performance in service, timber connections have been studied widely [13,14,15,16]. Eurocode 5 part 1-1 [17] provides simplified equations for calculating the load-carrying capacity of a single-shear and double-shear plane in different connection types, and double-shear timber connections with steel fasteners have been used in heavy wood structures [18]. Fonseca et al. [19] presented methodologies to evaluate the safe design of three types of timber connections in double shear, which was proposed by Eurocode 5, part 1-1, and analyzed the influence of the design parameters. Creep is an important characteristic of wood, so it is of great significance to study its deformation development law. Scholars have discussed the mechanisms of internal and external influences on wood creep from factors such as load size, temperature and humidity, tree species, repeated load, and load type. G. Hunt et al. [20] summarized a set of methods for predicting long-term creep of timber through short-term creep test data, which has been widely considered and applied. Yazdani et al. [21] conducted a full-scale model test on 16 T-section glulam simply supported beams, and obtained parameters closely related to the Burger creep model. Guo et al. [22,23] carried out long-term loading tests and short-term flexural tests on pure glulam beams, internally reinforced glulam beams, and internally reinforced prestressed glulam beams, and systematically analyzed the influence of factors such as loading level, reinforcement rate, and prestress value. In-depth research and discussion have been carried out on glulam simply supported beams with different cross-section and reinforcement forms, and the creep basic theory. It has provided a reference for this study in terms of long-term test methods and considerations, but a reinforcement method for external prestressed steel wires and the long-term performance research on continuous beams are still lacking, which indicates the necessity of carrying out long-term tests on prestressed glulam continuous beams.

In this paper, a total of 10 prestressed glulam continuous beams were subjected to a 180-day long-term loading test. By comparing the changes in steel wire stress and mid-span long-term deflection over time under the influence of creep, the effects of the number of prestressed steel wires and the prestress level on the long-term mechanical performance of prestressed glulam continuous beams were studied. Three models were selected to fit the creep process of the test beams. The creep deformation coefficient that reflected the long-term deflection growth of the test beams was determined, which lay the foundation for establishing the long-term deflection calculation formula for the prestressed glulam continuous beam.

## 2. Materials and Methods

### 2.1. Materials and Specimens

The same batch of spruce materials was used to produce the prestressed glulam continuous beams and the small clear specimens. 1860-grade, 7 mm diameter low-relaxation prestressed steel wires were recommended for all wire. Based on Chinese standards GB/T50329-2012 [24], GB/T1938-2009 [25], and GB/T228.1-2010 [26], material properties experiments were conducted at the laboratory of Northeast Forestry University. The relevant mechanical properties of each material as shown in Table 1.

**Figure 1:**
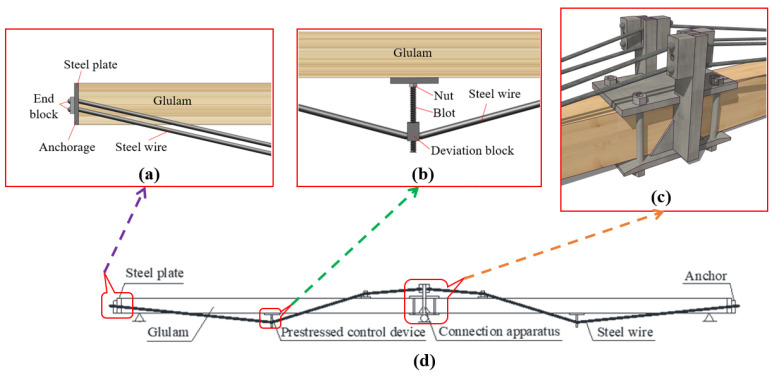

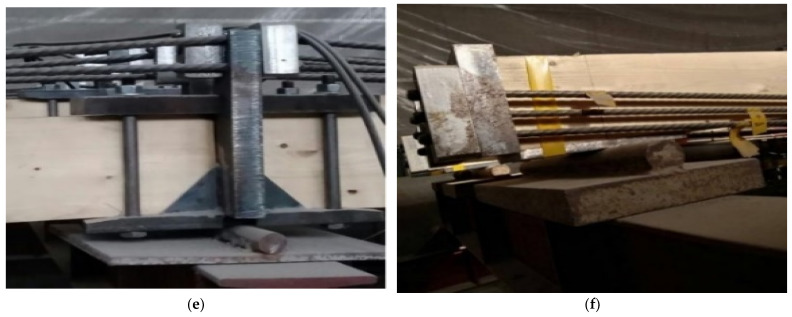
Diagrams of prestressed glulam continuous beam: (**a**) steel plate and anchor, (**b**) prestressed control device, (**c**) connection apparatus, (**d**) details of specimen, (**e**) photo of the connection apparatus, and (**f**) photo of the steel plate and anchor.

The prestressed glulam continuous beam was composed of glulam, high-strength steel wires, and steel components, as shown in Figure 1. The steel components included connection apparatuses, prestressed control devices, steel plates, and anchors. Five pieces of 20 mm-thick spruce sheets bonded together to form glulam with dimensions of 3080 mm × 80 mm × 100 mm (length × width × height). Therefore, the size of the prestressed glulam continuous beam was 6160 mm × 80 mm × 100 mm (Length × width × height). All the specimens were designed by the Chinese standard GB50005-2017 [27] and Canadian standard CSA O86-14 [28].

To study the effect of the number of steel wires and prestress level on the long-term performance of prestressed glulam continuous beams, the test beams were divided into two groups, A and B. Group A was used to research the effect of the number of steel wires, the first number represents the different number of steel wires, the second number indicates that there are two test beams under the same condition. Group B was used to research the effect of prestress level; the first number represents the different prestress values, and the second number indicates that there are two test beams under the same conditions. More grouping details are shown in Table 2. It is worth noting that the prestress was realized by turning the nut counterclockwise caused the screw to rotate, as shown in Figure 1b. The deviation block moved downward, and thus, the reinforcements were stretched, and the prestress was applied to glulam beams. By adjusting the distance from the deviation block to the bottom of the beam, the value of prestressing can be controlled.

### 2.2. Long-Term Test

#### 2.2.1. Loading Device and Method

**Figure 2:**
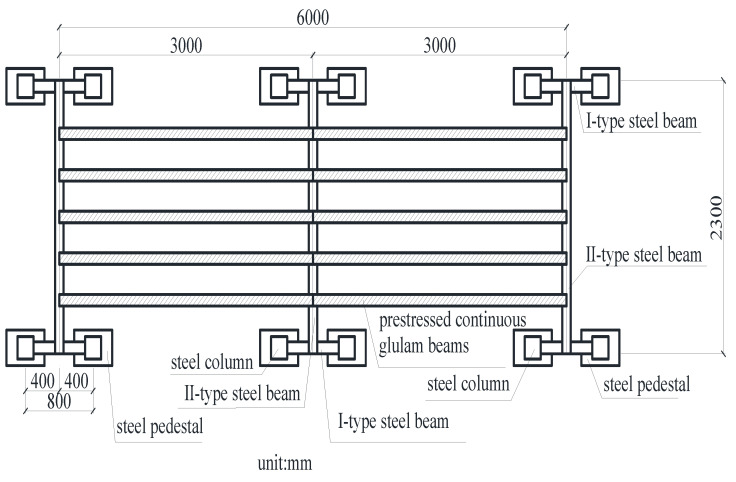
The plane layout of the test device.

**Figure 3:**
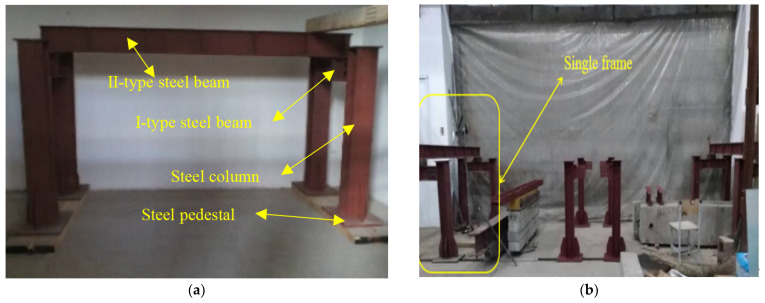
Long-term loading test device. (**a**) The composition of the single-loaded frame; (**b**) the whole loading support.

A long-term loading test device was designed by our research team, the plane layout and object pictures of the device are shown in Figure 2 and Figure 3. The device was composed of three identical parts, and each part includes four steel columns, two I-type steel beams, and one II-type steel beam. The steel column and I-type steel beams are connected by prefabricated bolt holes to form foundation support, and then the II-type steel beam is placed on top of the two foundation supports. The device can be used to carry out long-term tests on five beams at the same time, with high efficiency. The device has a large bearing capacity and rigidity and has little influence on the test results. In addition to this, it also can adjust the distance between two parts according to the length of the test beams, which has good applicability.

**Figure 4:**
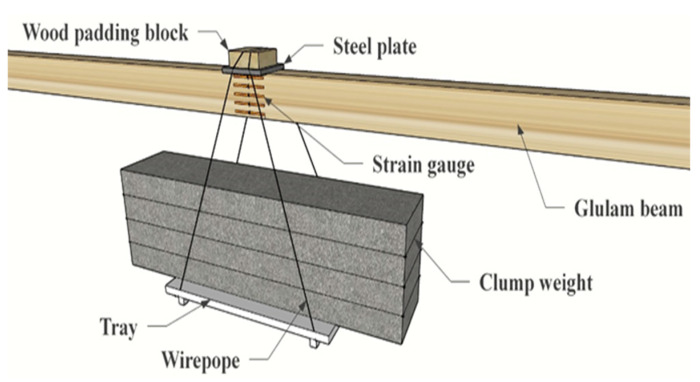
Loading method.

The test adopted the form of four-point bending for long-term loading. To ensure the constant loading value during the loading period, the long-term load was applied by hanging the corresponding weight in the midspan of the left and right spans of the continuous beam, respectively. Two steel plates were placed on the upper surface of the mid-span position of the left and right spans, and a small wooden block was placed on each steel plate, which prevents local compression failure at the loading point. Single-span loading is shown in Figure 4.

#### 2.2.2. Determine Long-Term Loads and Times

**Figure 5:**
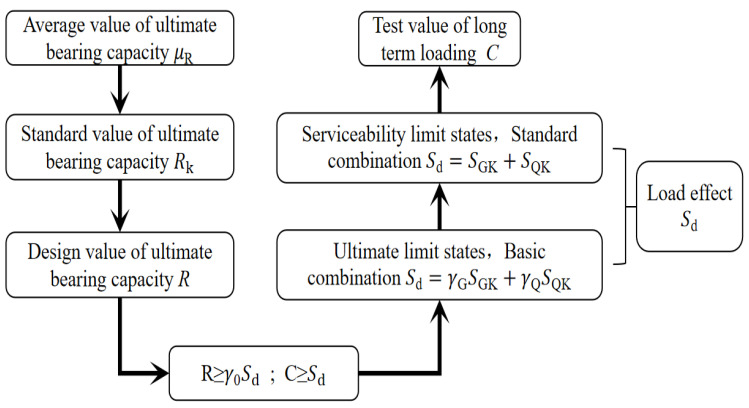
Derivation and determination of long-term loading value.

According to the Chinese standard GB50009-2012 [29] for the derivation process of the design value of the bearing capacity of the flexural member and the relevant content of the load effect combination. In terms of ultimate bearing capacity, the average value of the ultimate bearing capacity of the beams (*μ*_R_) was obtained based on the short-term bending experiment data of prestressed glulam continuous beams conducted by the research group in the early stage [12]. The standard value of the ultimate bearing capacity (*R*_k_) under certain reliability was obtained through the probability distribution, and then the design value (*R*) was obtained through the resistance partial coefficient. In terms of load effects, the load effects (*S_d_*) of prestressed members under the serviceability limit state and the ultimate limit state were calculated based on the ratio of dead load and live load effect of floor and office buildings was 2:3. The serviceability limit state shall adopt the standard combination, and the bearing capacity limit state shall adopt the basic combination. The process is shown in Figure 5. It is calculated that the normal service load of the beam was 0.33 times its ultimate bearing capacity. According to the tests performed by previous researchers, the loading level range is generally 30–70% of the ultimate failure load [30], so the long-term load value was determined to be 30% of the ultimate bearing capacity obtained from the short-term loading test. By consulting Chinese standards [31] and referring to previous experience [32,33,34,35], the long-term loading time of the beam was determined to be 180 days.

#### 2.2.3. Measurement Layout

**Figure 6:**
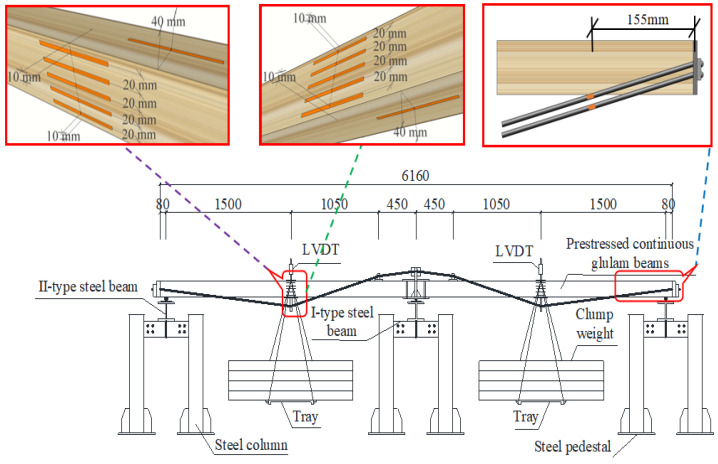
Test setup and measuring point layout of the long-term loading test.

Test measurements include beam mid-span displacement, glulam beam strain, and steel wire strain. In order to monitor the change in beam mid-span deflection with time, two LVDTs with a range of 150 mm were set in the midspan of the left and right spans. The use range of the LVDT used in the test was between 20% and 80% of the total range, and the LVDT connected to the line was calibrated and placed on the upper surface of the middle span of the test beam through the magnetic seat fixed on the support. To measure the strain of the glulam beams, five 100 mm × 3 mm strain gauges were pasted at equal intervals on the mid-span sides of the left and right spans of the continuous beam, a strain gauge was pasted at the beam top and bottom of the mid-span of the left and right spans, respectively. To measure the strain of the steel wires, the 2 mm × 3 mm strain gauges were pasted to the steel wire surfaces at a distance of 155 mm from the support points at both ends of the beam, the steel wires were polished by an angle grinder in advance. The specific details are shown in Figure 6.

First, the beam was applied to prestress, and at the same time, JM3813 static strain acquisition systems (Yangzhou Jing Ming Technology Co., Ltd., Yangzhou, China) were applied to collect the strain and deflection measurements synchronously. The professional Shield wire was used to connect JM3813 static strain acquisition box with LVDT and strain gauge, and in order to make the collected data more accurate, glulam, steel wire and strain acquisition box had connection compensation wire. Secondly, after the beam reached the expected prestress value, the heavyweight was hung to supply the load. According to the development law of the long-term deformation of the beam, the time interval between two data collections gradually increases as the experiment continues. The method of man-machine combination reading data was used, the details of the data collection arrangement are shown in Table 3.

## 3. Test Results and Analysis

### 3.1. Change in Temperature and Relative Humidity

According to the previous research results [36], the external environment (temperature and moisture content) can obviously affect the creep deformation of wood chipboard under bending. With the increase in the temperature and relative humidity, the creep deformation of wood would increase. In this study, the whole long-term loading process was carried out under normal indoor environment in Harbin, and test beams were divided into two batches and loaded in the same season. During this period, measures such as sprinkling water in summer and heating in winter were taken, and the temperature and humidity of each batch of the beams were regularly monitored so that the relative stability of the indoor environment could be ensured, so the influence of the change in the temperature and humidity on the analysis of the test results could be ignored.

### 3.2. Test Phenomenon

**Figure 7:**
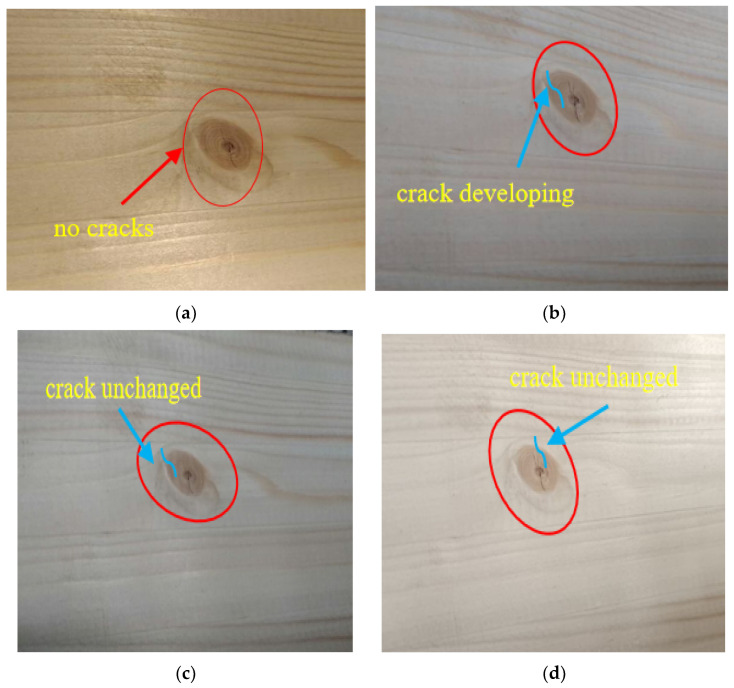
Long-term loading phenomenon of prestressed glulam continuous beam. (**a**) Test for 1 day; (**b**) test at 60 days; (**c**) test at 120 days; (**d**) test at 180 days.

On the first day, the 60th day, the 120th day, and the 180th day of the long-term test, the changes in the test beam were observed, as shown in Figure 7. Compared with the first day of the experiment, cracks at the wood-knot position of the test beam expanded slightly on the 60th day of the test and did not expand on the 120th and 180th days. Since the load applied in the long-term test was equivalent to the normal service load of the beam, and the force state of the beams under normal service condition were simulated, there were no obvious damage phenomenon such as cracks and wrinkles, except that the deflection of the test beam had obvious changes.

### 3.3. Stress Variation Law of Prestressed Steel Wire

Based on the previous experiments, it has been shown that when the initial stress of the prestressed steel wire was less than 0.5 times the value of the ultimate tensile strength R_y_, the influence of the relaxation of the steel wire could be ignored [37,38,39,40,41,42]. The maximum initial prestress applied to the steel wire in this test was 0.3R_y_ (0.3R_y_ < 0.5R_y_). Therefore, the influence of the relaxation of the prestressed steel wires(0.3R_y_) could be ignored, which indicates the creep of the glulam results in the main stress loss of steel wires.

**Figure 8:**
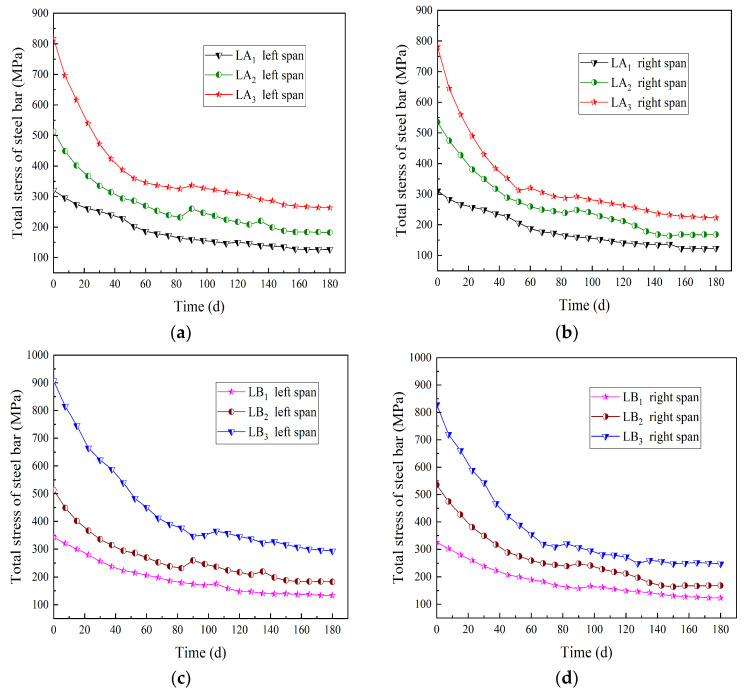
Time-varying curve of total stress of the steel wires. (**a**) Left span of group A (different steel wires quantities); (**b**) right span of group A (different steel wires quantities); (**c**) left span of group B (different prestress values); (**d**) right span of group B (different prestress values).

For the beams in groups A and B, the time-varying curve of the total stress of the steel wire is shown in Figure 8. The total stress means the stress sum of all steel wires in a test beam. The time process is from the initial loading to the end of loading. For the continuous beams, the stress changes for left and right spans were plotted separately.

It is known from Figure 8 that for the beams in group A, when the prestress value was unchanged and the number of prestressed steel wires was 2, 4, and 6, respectively, from the beginning to the end of the loading, the total stress values of the steel wires decreased by 60.39%, 66.49% and 69.61%, respectively. For the beams in group B, when the number of prestressed steel wires was unchanged and the prestress values were 0, 7, and 14 kN, respectively, from the beginning to the end of the loading, the total stress values of the steel wires decreased by 61.60%, 66.53%, and 68.74%, respectively. It is worth noting that the total stress values were the average values of the left span and the right span of the same continuous beam. Due to the increase in the number of prestressed steel wires or the prestress value, the external load on the continuous beam increased, then the section stress of the glulam also increased, and the wood creep was more obvious, and the total stress of the steel wires decreased more.

**Figure 9:**
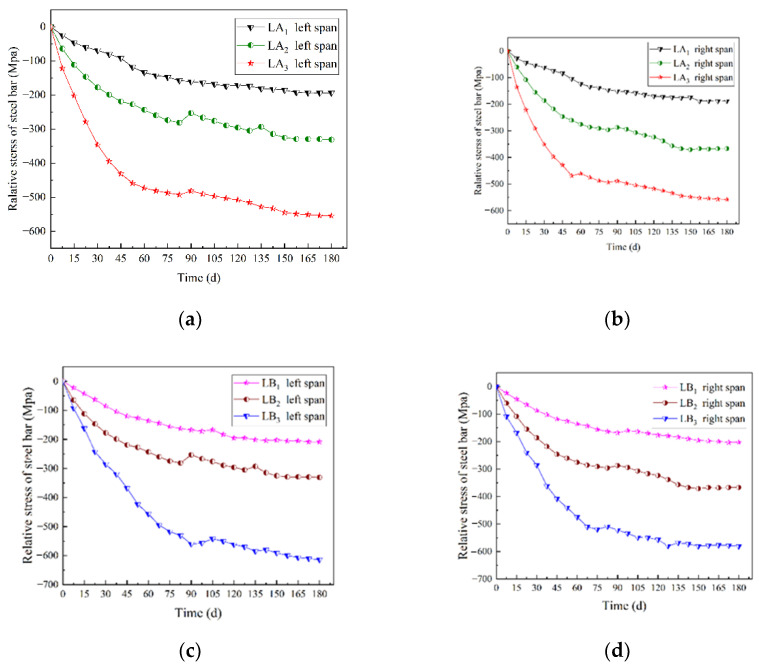
Time-varying curve of the relative stress of the steel wires. (**a**) Left span of group A (different steel wires quantities); (**b**) right span of group A (different steel wires quantities); (**c**) left span of group B (different prestress values); (**d**) right span of group B (different prestress values).

In order to study the influences of the number of prestressed steel wires and the prestress level on the change rate of the steel wire stress value, the initial stress of the steel wire was set to 0, and the variation of steel wire stress value during the experiment was defined as the relative stress value of the steel wire. The time-varying curve of the relative stress of the steel wire is shown in Figure 9.

It can be seen from Figure 9 that for the magnitude of the relative stress change, LA_2_ is 1.71 times larger than LA_1_, and LA_3_ is 2.87 times larger than LA_1_; in addition, LB_2_ is 1.58 times larger than LB_1_, and LB_3_ is 2.94 times larger than LB_1_. The relative stress of the left and right spans of continuous beams changes with time in the same law. This shows that by increasing the number of prestressed steel wires and the applied prestress value, the relative stress value increased, the total stress of the steel wires declined faster, and the effect on the creep of glulam was more obvious.

### 3.4. Variation Law of Beam Mid-Span Deflection

**Figure 10:**
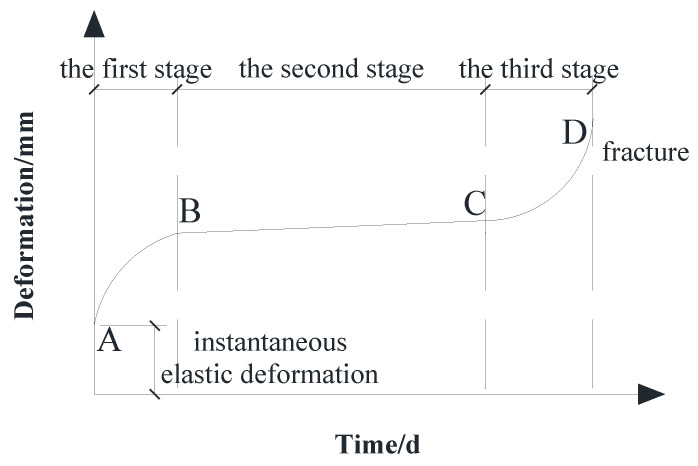
Diagram of the typical creep curve.

The typical creep curve is shown in Figure 10. It can be divided into three stages: the first stage is the transient creep, which means that the wood is applied with a certain load, and produces an instantaneous deformation. The deformation rate of this stage is continuously reduced until it reaches a relatively stable state. The second stage is the steady-state creep, the rate of deformation remains basically unchanged. That is, at the same time, the deformations are approximately equal. This stage lasts the longest and is the most typical during the entire creep development process. The third stage is the accelerated creep, where the deformation rate increases suddenly until the material damages.

**Figure 11:**
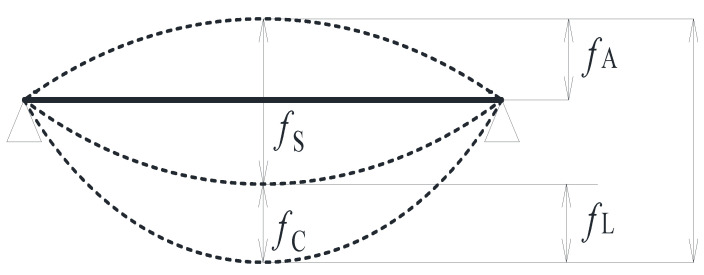
Deformation diagram of the prestressed glulam continuous beam during loading.

During the long-term loading test, the total deflection of the continuous beam is divided into three parts: the first part is the height of anti-arch *f*_A_ of the beam when the prestressing is applied, the second is the instantaneous deflection of the beam when the hanging weight provides an instantaneous load, and the third is the additional deflection *f*_C_ caused by the creep of wood under continuous long-term loading. It is worth mentioning that the deflection of the first and second parts are identified as short-term deflection *f*_S_, and the deflection of the third part was identified as long-term deflection *f*_L_. The sum of the three parts was the total deflection, as shown in Figure 11. The downward deflection is specified to be positive and the upward deflection to be negative. Due to the initial prestress was applied, the beams is reversed and the initial value of mid-span deflection was negative. The short-term, long-term, and total deflection values of beams of A and B in the test are shown in Table 4.

**Figure 12:**
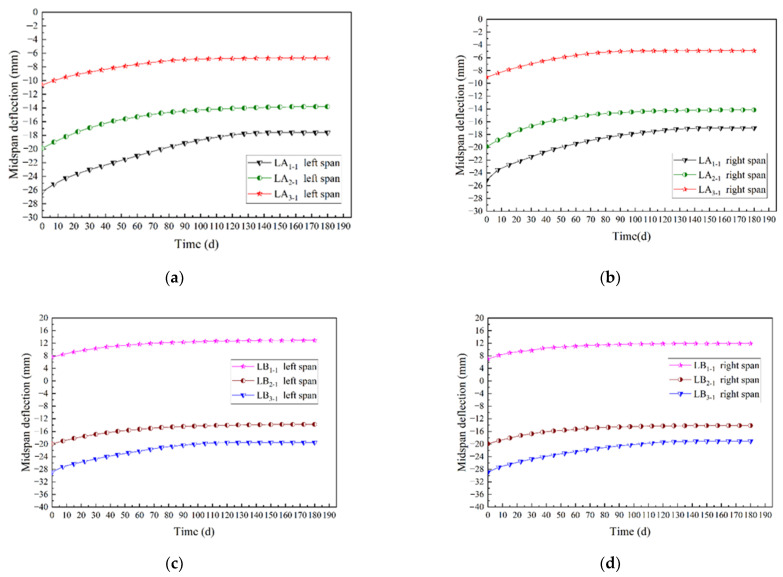
Time-varying curves of midspan deflection: (**a**) Left span of group A (different steel wires quantities); (**b**) right span of group A (different steel wires quantities); (**c**) left span of group B (different prestress values); (**d**) right span of group B (different prestress values).

To explore the creep law of prestressed glulam continuous beams under long-term loading, the time-varying curve of midspan deflection of group A and B are drawn, as shown in Figure 12.

It can be seen from Figure 10 and Figure 12 that the time-varying curve of midspan deflection of the continuous beam is close to the typical creep curve. For group A, the midspan long-term deflection of the beams had reached 60.8% to 85.0% of the total deflection on the 60th day, and the deflection at the 120th day had reached 96.1% to 99.8%; for group B, the midspan long-term deflection of the beams had accomplished 66.1% to 80.1% of the total deflection on the 60th day, and the deflection at the 120th day had accomplished 95.7% to 99.0%. It could be seen that the beam completed the primary creep faster and the creep rate decreased with time, which was consistent with the characteristic of the creep development of glulam. It is worth noting that LB1 was not applied prestressing. Therefore, the beam had a downward initial deflection after loading, and the initial deflection value was positive. In other cases, due to the application of different prestressing, the beams produced an upward anti-arch with a negative initial deflection value.

**Figure 13:**
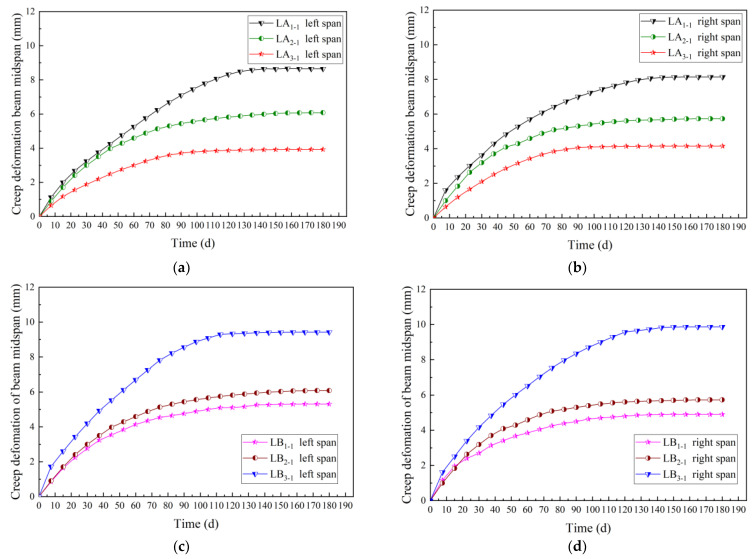
Time-varying curves of the creep deformation: (**a**) left span of group A (different steel wires quantities); (**b**) right span of group A (different steel wires quantities); (**c**) left span of group B (different prestress values); (**d**) right span of group B (different prestress values).

To analyze the influences of the number of prestressed steel wires and the prestress level on the long-term creep rate of the continuous beams in the mid-span, the short-term deflection was ignored, which was assumed that the deflection of the beam was zero after instantaneous loading, then the time-varying curve of the creep deformation is gained and shown in Figure 13.

It can be seen from Figure 13 that for the beams in group A, compared with the continuous beam with two steel wires, the long-term deflection average of the beams with four and six steel wires decreased by 32.0% and 48.0%, respectively. It showed that the long-term deflection gradually decreased with the increase in the steel wires. Therefore, increasing the steel wire could effectively reduce the long-term deflection of the prestressed glulam continuous beam. For the beams in group B, compared with the continuous beam without prestressing, the long-term deflection average of the beams with 7 kN and 14 kN prestress values increased by 18.75% and 88.40%, respectively. It could be seen that the long-term deflection grew gradually with the increase in the applied prestress level. Because the anti-arch value of the beam increased with the applied prestress level, resulting in more stress on the beam, the creep deformation of the glulam was more significant.

For group A, due to the number of steel wires increased, the growth rate of long-term deflection gradually slowed down. Increasing the number of steel wires can effectively restrain the long-term deflection change rate of the continuous beams; for group B, with the increase in applied prestress level, the growth rate of long-term deflection sped up. Therefore, increasing the prestress value could make the creep of glulam beams more obvious, promote the change rate of the long-term deflection of the continuous beams, and cause the deformation to develop rapidly.

### 3.5. Theoretical Analysis

#### 3.5.1. Establishment of Creep Model

In order to research the creep law of the test beams with time, the existing long-term test data is applied to establish creep model. According to previous experience [30], the prediction models for wood creep are as follows:

(1) $$y=a\left(1-e^{f\left(t\right)}\right)$$

Equation (1) shows the Exponential Equation [43]. Where *y* is the strain of material or deformation of component, *t* is the time when the member occurs creep deformation, *a* is a test parameter which is obtained by fitting the curve, and *f*(*t*) is a function related to *t*.

(2) $$y=a+bx^{c}$$

Equation (2) shows the power-law equation [44,45]. Where *y* is the strain of material or deformation of component, *x* is the time when the member occurs creep deformation, and *a*, *b*, and *c* are the test parameters that are obtained by fitting the curve.

(3) $$y=a+bt-ct^{2}$$

Equation (3) shows the Polynomial Equation [46]. Where *y* is the strain of material or deformation of component, *t* is the time when the member occurs creep deformation, and *a*, *b*, and *c* are the test parameters that are obtained by fitting the curve.

**Figure 14:**
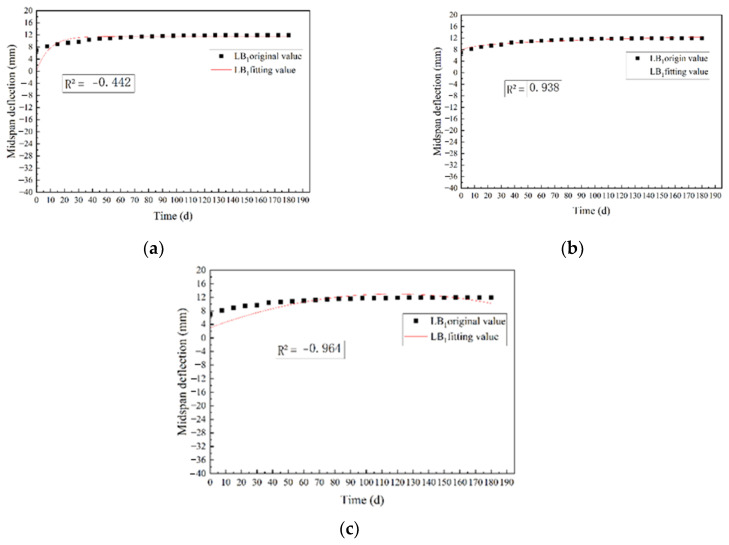
Midspan deflection fitting curve of LB_1_ beam under three models: (**a**) exponential equation fitting curve; (**b**) power-law equation fitting curve; (**c**) polynomial equation fitting curve.

Based on the three models, the mid-span deflection variation of the test beam LB_1_ under long-term loading was simulated, and the results are shown in Figure 14.

For the fitted curves, R^2^ reflects the fitting degree of the regression equation, and expresses the relationship between the dependent variable and all independent variables, it means the percentage of the dependent variable whose variability can be explained by the regression equation. The fit result is better with the increase in the R^2^ value, R^2^ is represented by Equation (4):

(4) $$R^{2}=1-\frac{\mathrm{RSS}}{\mathrm{TSS}}$$

where R^2^ is the coefficient of determination, RSS is the sum of squared differences between the fitted data and the original data, and TSS is the sum of squared differences between the original data and the mean value.

Research shows that the most commonly used empirical model is the power-law model [46,47]. It can be seen from Figure 14 that the determination coefficients R^2^ of the creep model of the power-law equation, exponential equation, and polynomial equation were 0.938, −0.442, and −0.964, respectively. The comparison results show that the power-law equation could effectively predict the creep process, and the model is simpler and more effective than the other two. Therefore, the power-law equation was selected to fit the midspan deflection of the test beams, the fitting parameters *a*, *b*, *c*, and the determination coefficient R^2^ were shown in Table 5.

It can be seen from Table 5 that all the R^2^ values obtained by fitting the test beams are greater than 0.9, indicating that the fitting accuracy of the mid-span deflection results and the fitting curve is high, and the creep behavior of the prestressed glulam continuous beams was shown preferably.

#### 3.5.2. Creep Deformation Coefficient *θ* and the Total Deflection

Three types of allowable value and calculation formulas were given for the deflection of flexural members in Eurcode5 [17]. Three types of allowable values contain instantaneous deflection *ω*_inst_, final total deflection *ω*_fin_, and final net deflection *ω*_net, fin_. The calculation of the total deformation of the flexural members is also specified clearly in American standards NDS-1997 [48]. According to the above standards, the creep deformation coefficient *θ* is defined as the ratio of the additional deflection produced by creep and the elastic deflection produced by loading in this paper. It is expressed by Equation (5):

(5) $$\theta =\frac{f_{C}}{f_{S}}=\frac{f_{L}}{f_{S}}$$

where *θ* is the creep deformation coefficient, *f*_c_ is the additional deflection value produced by the creep of the test beam, *f*_s_ is the elastic deflection produced by the load (the short-term deflection value of the prestressed glulam continuous beam), and *f*_L_ is the long-term deflection value of the prestressed glulam continuous beam.

The total deflection of flexural members under normal service conditions includes the elastic deflection caused by loading and the creep deflection caused by the creep of wood under continuous load. The elastic deflection *f*_S_ of the flexural members could be calculated according to structural mechanics, while the creep deflection could be calculated by multiplying the deformation *f*_Q_ by the creep deformation coefficient *θ*. The calculation formula was shown in Equation (6):

(6) $$f_{T}=f_{S}+\theta f_{Q}$$

where *f*_T_ is the total deflection of the test beam, *f*_Q_ is the deformation due to quasi-permanent combination of loads.

Because the service life of the building is generally 50 years, the power-law model is used to predict the total deflection value of the beams for 50 years in this paper, and the creep deformation coefficient of the test beams is calculated by Equation (5), the results are shown in Table 6. Table 6 shows the value range of creep deformation coefficient was 1.28–2.29 when the steel wires on the beam were from 2 to 6, and the *θ* value decreased with the steel wires increased; the value range of creep deformation coefficient was 1.69–2.17 when the prestress was from 0 to 14 kN, and the *θ* value decreased with the prestress increased. Therefore, the creep deformation coefficient *θ* was determined to be 1.28–2.29 in the range of familiar steel wire quantities and applied prestress.

## 4. Conclusions

- When the number of the prestressed steel wires was 2, 4, and 6, the total stress of steel wires decreased by 60.39%, 66.49%, and 69.61%, respectively, from the beginning to the end of loading; when the prestress value was 0, 7, and 14 kN, the total stress of steel wires decreased by 61.60%, 66.53%, and 68.74%, respectively, from the beginning to the end of loading. This is because by increasing the number of prestressed steel wires and the prestress value, the external load on the continuous beam increased, then the section stress of the glulam also increased, and the wood creep was more obvious, and the total stress of the steel wires decreased more.
- Compared with the continuous beam with two steel wires, the long-term deflection average of the beams with four and six steel wires decreased by 32.0% and 48.0%, respectively; compared with the continuous beam without prestressing, the long-term deflection average of the beams with 7 kN and 14 kN prestress value increased by 18.75% and 88.40%, respectively, which shows that increasing the number of steel wires could effectively restrain the long-term deflection change rate of the continuous beams, and increasing the prestress value made the creep of glulam beams more obvious and promoted the long-term deflection change rate of the continuous beams, the deformation developed faster.
- Based on the comparison between three common creep models of exponential equation, power-law equation, and polynomial equation, it found that the power-law equation was the most suitable for simulating the creep process of the prestressed glulam continuous beams, and the fitting curve had higher accuracy. According to the experiment and numerical fitting results, at familiar steel wire quantities and prestress levels, the creep deformation coefficient of the test beams within the design service life of 50 years was determined to be 1.28–2.29.
- The calculation formula of long-term deflection and creep deformation coefficient of test beams were given by long-term experiment and model fitting. Later, the accuracy of fitting can be verified by longer longer-term experiments.
- The long-term performance of prestressed glulam continuous beams was mainly studied from the perspective of experiment in this paper. In the future, its long-term constitutive relation can be exploited by finite element analysis.

## Figures and Tables

**Table 1:** Material properties.

Material	Characteristics	Average (N/mm^2^)	COV (%)
Spruce	Modulus of elasticity in compression	8.301 × 10^3^	4.630
	Compression strength parallel to grain	38.67	6.151
	Modulus of elasticity in tensile	9.750 × 10^3^	6.42
	Tensile strength parallel to grain	81.32	6.184
Steel wire	Modulus of elasticity	1.92 × 10^5^	1.710
	Tensile strength	1.640 × 10^3^	0.354

**Table 2:** Groups and information of specimens.

Group	Number of the Component	Prestress Value (kN)	The Number of Steel Wire	Long-Term Loading Value (kN)	Loading Time (day)
A	LA_1-1_	7	2	8.24	180
	LA_1-2_	7	2	8.92	
	LA_2-1_ *	7	4	9.24	
	LA_2-2_ *	7	4	9.24	
	LA_3-1_	7	6	10.06	
	LA_3-2_	7	6	9.55	
B	LB_1-1_	0	4	8.24	180
	LB_1-2_	0	4	8.92	
	LB_2-1_ *	7	4	9.24	
	LB_2-2_ *	7	4	9.24	
	LB_3-1_	14	4	10.06	
	LB_3-2_	14	4	9.55	

Note: “*” indicates that LA_2-1_ and LB_2-1_ in the table are the same beams, but they are in different groups, so different subscript characters are labeled. LA_2-2_ and LB_2-2_ are the same situation.

**Table 3:** Information on data collection interval.

Test Time	Collection Time Interval	Manual Data Collection Time Interval
Days 1~4	0.25 h/time	0.25 h/time
Days 5~12	0.25 h/time	0.5 h/time
Day 13~20	0.5 h/time	1 h/time
Day 21~28	0.5 h/time	2 h/time
Day 29~56	1 h/time	4 h/time
Day 57~180	2 h/time	12 h/time

**Table 4:** Midspan deflection value of prestressed glulam continuous beams.

Beam Number		Short-Term Deflection *f*_S_ (mm)	Long-Term Deflection *f*_L_ (mm)	Total Deflection *f*_T_ (mm)
LA_1_	Left span	−26.61	8.70	−17.91
	Right span	−25.35	8.42	−16.93
LA_2_	Left span	−20.03	6.00	−14.03
	Right span	−20.09	5.64	−14.45
LA_3_	Left span	−10.18	4.33	−5.85
	Right span	−10.28	4.56	−5.72
LB_1_	Left span	7.51	4.93	12.44
	Right span	7.60	4.87	12.47
LB_2_	Left span	−20.03	6.00	−14.03
	Right span	−20.09	5.64	−14.45
LB_3_	Left span	−29.21	8.97	−20.24
	Right span	−28.87	9.49	−19.38

**Table 5:** Result of fitting parameters.

Beam Number	*a*	*b*	*c*	R^2^
LA_1_	−29.24	3.04	0.28	0.96
LA_2_/LB_2_	−26.49	6.18	0.34	0.96
LA_3_	−14.75	2.82	0.22	0.94
LB_1_	4.04	12.77	0.09	0.97
LB_3_	−30.71	3.11	0.42	0.98

Note: LA_2_ and LB_2_ in the table are the same beams, but they are in different groups, so different subscript characters are labeled.

**Table 6:** Creep deformation coefficient for 50 years.

**Project**	**Group A**			**Group B**		
	**LA_1_**	**LA_2_**	**LA_3_**	**LB_1_**	**LB_2_**	**LB_3_**
Short-term deflection *f*_s_ (mm)	6.12	5.53	3.54	7.01	5.53	5.44
50-year total deflection *f*_T_ (mm)	20.15	15.38	8.08	22.20	15.38	14.63
Creep deformation coefficient *θ*	2.29	1.78	1.28	2.17	1.78	1.69

## Data Availability

The data presented in this study are available on request from the corresponding author.
